# Comprehensive care for people affected by TB: addressing TB-associated disabilities

**DOI:** 10.5588/ijtldopen.24.0167

**Published:** 2024-05-01

**Authors:** M. Calvi, D. Boccia, E. Jaramillo, F. Mavhunga, J. Reeder, T. Kasaeva

**Affiliations:** ^1^ Global Tuberculosis Programme, World Health Organization (WHO), Geneva, Switzerland;; ^2^ Department of Global Health and Development, Faculty of Public Health and Policy, London School of Hygiene & Tropical Medicine, London, UK;; ^3^ Special Programme for Research and Training in Tropical Diseases (TDR), WHO, Geneva, Switzerland

**Keywords:** COPD, rehabilitation, tuberculosis, people-centred care, survivors, PTLD, social protection

In the global fight against TB (the second leading cause of global deaths due to an infectious agent),^1^ one fact remains stark: despite 75 million lives having been saved since 2000,^2^ too many TB survivors are left with lasting impairments. These consequences, when coupled with some TB risk factors, result in impairments and disabilities affecting their overall quality of life, life expectancy and economic productivity. TB-associated impairments and disabilities can be a direct consequence of the disease and/or its treatment and can encompass a spectrum of health conditions affecting mostly pulmonary, musculoskeletal, and mental functions.^3^ Pulmonary TB often exacerbates pre-existing chronic conditions, such as chronic obstructive pulmonary disease (COPD), or results in bronchiectasis and pulmonary hypertension.^4^ Over the past decade, the study of post-TB impairment and disabilities has gained momentum and this is reflected in the growing literature on the topic^5^ to elucidate the burden,^6^ the risk factors,^7^ and the management of these conditions.^8,9^ Although the scientific and clinical aspects are being intensively investigated,^10–12^ few studies examine how TB-associated disabilities may be effectively detected and managed under programmatic conditions.^13^

This issue of the *Journal* includes an implementation research study by Adakun et al.^2^ (on behalf of the Kenya, Uganda, Zambia and Zimbabwe TB Disability Study Group), on the feasibility of assessing for comorbidities, impairments, disability, and determinants of disabilities among people successfully completing TB treatment and referring them for care in selected health facilities. The study was conducted in low-income settings with significant TB burden and confirms the impact of associated impairments and disabilities on TB survivors. It is a matter of major concern that one-quarter of people who completed TB treatment continued to suffer from ongoing respiratory symptoms and one in five had evidence of cardio-pulmonary impairment. Although in-facility referrals were excellent (>98%) for HIV co-infection and malnutrition, they appear to be sub-optimal for smoking, probable alcohol dependence, occupational exposure to silica and substance use. It is significant that 80% of patients with signs of cardio-pulmonary disability needed to be referred outside the original health facility.

The study addresses important operational questions. First, it shows that the assessment of comorbidities and disabilities, and their associated TB risk factors in TB survivors appears feasible under programmatic conditions and can become an essential part of people-centred care. However, this needs to be evaluated in other settings and contexts before informing global policies. Second, the referral to external services (i.e., outside of TB care facilities) seems insufficient to provide the necessary rehabilitation, counselling and social care support. Efficient, and sustainable approaches to deliver integrated social care and rehabilitation services at the primary healthcare level must be developed with urgency and creativity. This requires coordinated collaboration within and beyond the health sector to address several challenges, including infrastructure, qualified human resources and coverage with social protection. Third, the study focuses on disabilities among TB survivors. However, the road to addressing TB-associated disabilities requires a multistage approach that takes into account the natural history of TB, in such a way that the impairments and disabilities can be prevented or detected early and effectively managed as soon as the patient is enrolled in TB treatment, in addition to rehabilitation whenever required during and after treatment completion. Although cross-sectional studies such as this one by Adakun et al.^2^ play an important role in measuring the burden of TB-associated disabilities and answering operational questions about their management, they do not fully explore the long-term impact of disabilities on the people affected by TB. Longitudinal studies (e.g., TB Sequel or those with an even longer follow-up period^14^) are needed to address this limitation and improve understanding of the life trajectory and expectancy of people affected by TB-associated disabilities, especially children and adolescents. Finally, research is needed to improve our understanding of the way biological and social determinants operate and interact to produce impairments and disabilities in people affected by TB. Several studies suggest that post-TB symptoms interfere with work and education, leaving TB-affected households financially vulnerable 6–12 months after the completion of treatment.^15,16^ This impact on human capital is thus likely to perpetuate or even exacerbate the vicious cycle of TB and poverty, putting TB survivors at higher risk of further impoverishment and social exclusion, and consequently, making them increasingly vulnerable to future episodes of TB.

Social protection can play a pivotal role in providing people with TB-associated disabilities with a range of services during and after TB treatment completion,^17^ mitigating or even compensating for the impact of new social vulnerabilities created by the disability. Psychosocial support also based on counselling, psychological therapy and social care have been identified as some of the most important factors affecting quality of life among individuals likely to experience severe TB sequelae.^18^ Feasible and sustainable multisectoral approaches are urgently needed to ensure the inclusion of social protection in the programmatic response to TB-associated disabilities.

The WHO policy brief on TB-associated disability^3^ aligns with these reflections and calls for integrated people-centred care, based on the prevention and early identification of impairments and disabilities through systematic screening and the implementation of effective referral mechanisms, as well as the development of coordinated multidisciplinary models of care within and beyond the health sector. The translation of these policy recommendations into national programmatic actions for people with TB-associated disabilities requires a solid roadmap for research. The evidence emerging in recent years on the ways in which TB affects people during treatment and after being declared ‘cured’ in the post-TB disease phase suggests that the TB community may need to start seeing TB as an event with the potential to profoundly derail the life trajectory of the people affected, whose management transcends the boundaries of medical treatment alone.
